# Research in an intercultural context: mediator-investigators of epidemiological health studies, bridges between two worlds

**DOI:** 10.3389/fpubh.2024.1342140

**Published:** 2024-07-03

**Authors:** Leslie Alcouffe, Marc-Alexandre Tareau, Margot Oberlis, Antoine Adenis, Nicolas Vignier

**Affiliations:** ^1^Institut Santé des Populations en Amazonie, Centre Hospitalier de Cayenne, Cayenne, French Guiana; ^2^Centre Hospitalier de Cayenne, Inserm CIC1424, Cayenne, French Guiana; ^3^Université de Guyane, Cayenne, French Guiana; ^4^Association Mélisse, Cayenne, French Guiana; ^5^Croix Rouge Française, Cayenne, French Guiana; ^6^Hôpitaux Universitaires Paris Seine-Saint-Denis, Hôpital Avicenne and Jean Verdier, AP-HP, Bobigny, French Guiana; ^7^IAME, Inserm UMR 1137, Université Sorbonne Paris Nord, UFR SMBH, Bobigny, France; ^8^Institut Convergences et Migration, Aubervilliers, French Guiana

**Keywords:** health, mediation, community health worker (CHW), community-based healthcare, research, French Guiana, humans rights, good clinical practice—GCP

## Abstract

**Context:**

French Guiana is a French overseas department where very different linguistic, cultural and societal populations live together in a small area. Health issues and their specific epidemiological profiles call for research projects crossing several cultures. In this context, health mediators have a role to play in research. The aim of this study was to describe the perceptions, attitudes and opinions on research of mediators and researchers collaborating on research projects, and to describe the strengths and difficulties encountered during this cooperation.

**Methods:**

The Inter-med project was conducted in French Guiana between February 2022 and April 2023 on the base of semi-directive interviews with mediators, or researchers, all working in health research in the intercultural context of French Guiana. The socio-demographic characteristics of the participants were described. An inductive thematic analysis was carried out on all the interviews, and word occurrence analysis on certain themes. The information was triangulated with field coordination notebooks from two epidemiological surveys conducted in French Guiana between 2021 and 2022.

**Results:**

A total of 26 semi-structured interviews were conducted and 1,328 notebook pages analyzed. Mediation was described as an indispensable interface between the world of research and that of the population targeted by a survey. Mediators have a role to play at different stages of projects, in respect of good clinical practice, ethics and legislation. They act as interfaces between languages, concepts and representations. Their profession remains under-defined and under-dimensioned. The jobs offered are often precarious. Mediation work is emotionally costly, calls on soft skills and requires a combination of rigor and flexibility. All these aspects are implemented in the specific world of research, where there are common concepts and divergent perceptions. Researchers and mediators converge on a common goal: improving health.

**Conclusion:**

This study covers several aspects of the development and implementation of research projects. Respect for good clinical practice and people, transparency and data quality are redundant concerns, and this study touches on ethnocentrism, stigmatization and cultural representations. This study points out that the integration and recognition of mediators could be beneficial in research conducted in a cross-cultural context.

## Introduction

French Guiana is a French region in South America. The multicultural dimension of this territory is one of its constitutive and historical characteristics. French Guiana faces major demographic and migratory challenges (1). Migration to French Guiana over the last 7 decades has contributed to the recomposition of social groups and their relationships. Currently, some 40 languages are spoken in French Guiana (2). In French Guiana, many communities are united around shared histories, including the Businenge, people from Haiti, Suriname, Brazil and the Dominican Republic, people in transit from Syria and Afghanistan, native-born people, and people from hexagonal France (3, 4). Part of the migration is also linked to illegal gold mining (5).

Although health is perceived differently by each individual, it has a universal dimension. In French Guiana, this intercultural context is reflected in the care provided, and whatever their origin, caregivers are confronted with the notion of alterity (6). In this work, the definition of culture used is that proposed by the Mexico Declaration: “the set of distinctive spiritual, material, intellectual and emotional features that characterize a society or social group.” It includes the arts and letters, ways of life, fundamental rights, value systems, traditions and beliefs (7). The definition of context used is “the circumstances or events that form the environment in which something exists or takes place” (8). Representations of health and illness have an impact on how users behave in the face of care (9). They also concern the explanations given by sick people for the origins of their disorders or illnesses, their perception of the various therapeutic or diagnostic tools, and their representation of the medical corps (6). Research has shown that in French Guiana, the coexistence of different cultures has led to a pluralistic approach to health care. Information circulates, leading to the appropriation of traditional Guyanese botanical knowledge by migrants, for example. These elements are constantly evolving (4). Beyond representations, interculturality can be illustrated by communication difficulties, conflicts of values or divergent perceptions (10).

The role of the healthcare mediator is a key one in healthcare contexts involving interculturality or the accompaniment of populations distant from healthcare (11). According to the French National Health Mediation Program, mediation has demonstrated its beneficial effects on access to healthcare, particularly for vulnerable populations (12). According to the French Health Authority, the health mediator’s role is to act as an interface, facilitating access to the healthcare system and to rights for the most vulnerable members of the public, as well as raising awareness among the actors in this system of the obstacles encountered by users during their healthcare journeys. In this way, the mediator helps to forge links with the populations concerned, facilitates the coordination of care, and proposes health promotion initiatives (11). On an international scale, the missions entrusted to them may be similar to those of community workers, or community-based health agents. In French Guiana, the cultural richness of the territory and its particular epidemiological profile (whether infectious or non-infectious) mean that the role of mediators is particularly important.

This interface role is also of interest in health research, particularly in this multicultural context. In French Guiana, many public health concerns are concentrated in vulnerable populations. Research into these questions takes place in a particularly heterogeneous context, and it is essential to take into account the diversity of representations of the body, health and illness (13). Involving the people concerned at different levels creates a link between scientists, investigators and respondents. This link is essential to guarantee the success and quality of a study, and to limit bias (14, 15). Numerous studies in French Guiana, past, present and future, covering a wide range of fields, will involve, or have involved, health mediators. In France, research is regulated by the Jardé Law of 2012, which provides a framework for research and guarantees the security of individuals and their personal data (16). With the authorization of the French ethics authorities, mediators can take on the role of investigators in certain research projects, and are responsible for regulatory tasks. In French Guiana, it is crucial to rely on these resource persons as an interface between the world of academic research, and the field world of target populations. In order to capitalize on recent experiences in French Guiana, and to explore and understand the perception of interculturality and the place of mediation in research, the Inter-Med study (Health research in a context of Interculturality: the place of Mediators as skilled people) was conducted. The aim of this study was to describe the perceptions, attitudes and opinions of mediators and researchers on the participation of mediators in clinical health research; and to describe the strengths and difficulties encountered in health research projects based on this cooperation between 2021 and 2023. In clinical research, the full integration of mediators into research projects, by making their role permanent, recognizing their skills and supporting the current structuring of the mediator profession, could be beneficial to all research conducted with specific populations and, more generally, in an intercultural context.

## Materials and methods

### The inter-med project

The Inter-Med project was conducted in French Guiana between February 2022 and April 2023 using semi-directive interviews with mediators, or researchers, all working in the intercultural context of French Guiana.

Participants were surveyed in 2 groups, the “mediator” group and the “researcher” group. The inclusion criteria for the “mediators” group were: have participated as an investigator or mediator in a health-related research project, either on a salaried or volunteer basis; and for the “researchers” group: to have held a position as researcher, investigator or coordinator in a health research project involving mediators. The inclusion numbers of the “Researchers” group are composed as follows: 00-R and the inclusion numbers of the “Mediators” group are composed as follows: 00-M.

For the purpose of consistency, the interview guide varied a little according to the group surveyed, although the same themes were discussed. The themes explored were: the definition of research, its strengths and limitations, the importance attached to health surveys, aspects insufficiently taken into account by peers, definitions of the concepts of data, sample and representativeness. This was followed by a discussion of research experiences in collaboration with researchers for mediators, and in collaboration with mediators for researchers, the qualities required for the job of investigators, the definition of mediation, difficulties encountered in conducting studies, language barriers, the importance of cultural representations, personal perceptions of research and its evolution. These were in-depth individual interviews.

Two different interviewers conducted the interviews, in order to limit interviewer bias and ensure that, at the time of the study, the interviewer had no hierarchical link with the respondents. Interviews were conducted in French or in English, according to the respondent’s preference.

The sample is a convenience sample. People were surveyed until the information was saturated.

### The field coordination notebooks

Field coordination notebooks from two surveys were also used as study material: containing the daily notes of project coordination. The coordination notebook of the Guyasseremig study (Study on the Sexual and Reproductive Health of Migrant Women in French Guiana) was analyzed. This study is a cross-sectional epidemiological study, was conducted in French Guiana among migrant women between May 2021 and August 2021, mobilizing 2 mediators, at 2 inclusion points over 4 months.

The coordination notebooks from the Parcours d’Haïti study (Precarity and sexual vulnerability during the life course and migration of people from Haiti living with or without HIV in French Guiana) have been analyzed. This study is a cross-sectional epidemiological study, conducted in French Guiana, between November 2021 and May 2023, among people born in Haiti, mobilizing 7 mediators, in more than 30 inclusion points, over 22 months.

### Ethics statement

The Inter-med study is ancillary to the Parcours d’Haïti project authorized by the Committee for the Protection of Individuals South-East I under number CPP 2021–119, and was declared to the committee during the 3rd substantial amendment. The study has also been notified to the Health Data Hub. The respondents were given full oral and written information. The non-opposition form was signed by the investigator, in accordance with French legislation on non-interventional studies. Participants’ consent was sought for the audio recording of interviews. Individuals could withdraw from the study if they wished. A pseudonym number was assigned to each participant, and interviews were made anonymized.

### Analysis

Participant characteristics (age, level of education, gender and country of birth) were collected and described using percentages. The information was compiled in an Excel document and analyzed using Stata^®^ 16 software.

Recorded interviews from the Inter-Med project were transcribed, then analyzed in a synthetic inductive exploratory thematic way. This exploratory work consisted in gathering experiential knowledge, identifying and rearranging its themes, and coding the concepts. For the analysis, first, the interviews were listened to and transcribed, with a view to familiarization. The interviews were transcribed in the language in which they were conducted. Then, particularly salient and redundant themes were encoded. The themes were organized and discussed with an anthropologist familiar with interculturality and an experienced mediator who had not taken part in the survey. The titles of the themes were in French. Finally, the themes were finalized, and extracted in an excel document, with the associated verbatims. Following transcription and analysis, the titles and verbatims were translated into English. Those already in English have been used as is. The themes were summarized and written up. This work was carried out using MAXQDA2022^®^ software.

For the analysis of the qualities required to be an investigator in a health research project, lexicometric analyses were carried out, measuring the occurrence of terms. The qualities mentioned were recorded for each interview. If a quality was mentioned several times in the same interview, it was counted only once in terms of occurrence. The information was compiled in an Excel table, and the frequency of mention analyzed using Stata 16^®^. Synonymous words were grouped together under the heading “quality” for a given theme. Finally, the most frequently mentioned qualities (mentioned in more than 5 different interviews) were then analyzed by group.

For illustrative purposes, the data collected was triangulated from the Guyasseremig and Parcours d’Haïti survey notebooks, tracing the day-by-day coordination notes of these two projects. An inductive thematic analysis was conducted, highlighting the most salient themes and situations encountered in practice during the course of these two studies.

All the analysis presented below, written by a researcher has been reviewed by peer mediators, in order to limit interpretation bias.

Among the indicators chosen to guide the analysis, the key words used were the notions of research, data, sample and representativeness. The people targeted by the study are mostly French-speaking, so the definitions of “references” used were those of the Larousse French dictionary, associated with the context of science. Levels of education were grouped according to the International Standard Classification of Education (ISCED). For the analysis of intercultural skills, we used the definitions proposed by UNESCO.

## Results

In the Inter-med project, a total of 26 semi-structured interviews (each lasting between 15 and 140 min, depending on the participant) were analyzed. The socio-demographic characteristics of the participants were described (Table 1). One interview was conducted in English, and all the others in French. For the Guyasseremig survey, a 128-page notebook of daily notes taken between May and September 2021 was analyzed. For the Parcours d’Haïti survey, 1,200 pages of daily notes taken between September 2021 and July 2023 were analyzed.

Three main themes and 8 sub-themes emerged during the analysis: we will now present them one by one.

**Table 1:** Gender, region of birth, level of acamedic study, age of people interviewed in 2022 and 2023, by group, for the Inter-Med qualitative survey (*n* = 26).

	Researchers (*n* = 18)		Mediators (*n* = 8)	
	*n*	*%*	*n*	*%*
**Gender**	**18**	**100**	**8**	**100**
Women	11	61.1	7	87.5
Man	7	38.9	1	12.5
**Region of birth**	**18**	**100**	**8**	**100**
Hexagon France	16	88.8	0	0
Germany	1	5.6	0	0
Spain	1	5.6	0	0
French Guiana	0	0	1	12.5
Haiti	0	0	6	75
Brazil	0	0	1	12.5
**Level of academic study**	**18**	**100**	**8**	**100**
PhD or more *(8 years of university studies or more) ISCED 8*	12	66.7	0	0
Master’s degree *(5 years of university studies) ISCED 7*	5	27.8	0	0
Bachelor’s degree *(3 years of university studies) ISCED 6*	1	5.5	3	37.5
Advanced diploma *(2 years of university studies) ISCED 5*	0	0	1	12.5
High school degree *ISCED 4 and 5*	0	0	4	50
**Age**	*Median [IQR] n = 18*		*Median [IQR] n = 8*	
Age (Years)	36.5 [33–43]		37 [35–43.5]	

## First main theme: Health mediation, an indispensable interface between two worlds

### The involvement of mediators at different stages

**Figure 1:**
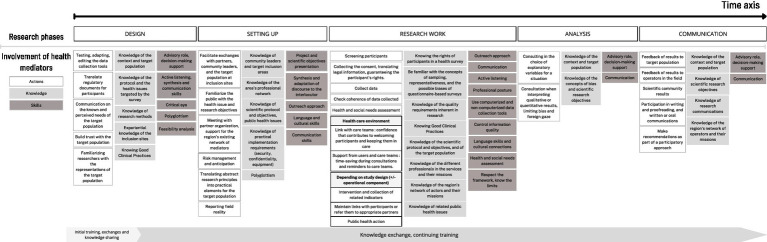
The different levels of mediator involvement in a health research project, based on research experience in French Guiana via the Inter-Med interviews, and on the coordination notebooks from the Guyasseremig and Parcours d’Haïti studies: actions, knowledge and skills.

The involvement of mediators according to the phase of the research projects are developed (Figure 1).

This multi-level involvement was highlighted as particularly important by a number of surveys. Mediators are sometimes described as the guarantors of feasibility and data quality. The importance of the mediators’ involvement in the early stages of project implementation was also raised, with a view to familiarizing them with the field, ensuring acceptability, encouraging adherence and respect. This preparatory work is carried out in both directions (with the population and with the research teams). However, this involvement right from the design stage is sometimes difficult to achieve, due to a lack of funding and therefore of mediation jobs.

“*I think it's something we can really improve, because research mainly comes from the university, private institutions, public institutions, but I think It should be closer to the real world.”* 025-R.

“*We have to draw up a protocol, we sometimes have to draw up an intervention, to be able to get regulatory approvals, to be able to get money, to be able to recruit people who will then take part but won't necessarily have taken part earlier, because until we have the money to pay them too, it's complicated, we haven't identified them.*” 026-R.

During surveys, the presence of mediator-interviewers ensures greater acceptance by the target population, while interpersonal skills and cultural decoding guarantee data quality and help to clarify certain answers.

“*The mediator can explain to us, afterwards, that when the question is asked, it's not very well put. There isn't really a shaman's diploma, but there are some people who call themselves shamans, but when we ask the question: have you been to see a shaman, or is there a shaman in the village? A person may very well answer "no", because in his or her eyes there is no real shaman in the village.”* 013-R.

### The role of mediators in good clinical practice, ethics and respect for legislation

The regulatory aspect and the administrative burden involved in setting up a research project were cited as one of the main difficulties or limitations, by the researchers’ group. In practice, legal documents such as the consent form or the non-opposition form are sometimes far removed from the vision of the people targeted by the study, for whom, for example, the notion of signature can be a source of questions. Given the complexity of this aspect, respondents report that some healthcare and research professionals are not very vigilant about its application.

“*People think that because they're doctors or whatever, what they're doing is necessarily good for people, and they're going to prejudge the level of information they're going to have to give people, or the way they're going to twist things up, and often it's a bit of a mascarade, the information notes, so yes, because people don't understand anything. But I don't think doctors take enough time to get down to the individual's level, to explain the ins and outs, to engage them in research.”* 001-R.

“*In other words, the authorizations and other things that are required of us today to be able to carry out research work are so restrictive, certainly with the aim of protecting people, but they are so restrictive...*” 010-R.

In this context, as research is subject to current legislation, mediators can act as a link, applying regulatory obligations in a way that is truly respectful of the understanding and rights of participants. Mediators translate information notes, and take the time to explain to participants their rights in a way that is intelligible to them, including the right to refuse to participate, the right not to respond, and the right to withdraw from the project and have their information deleted.

“*We know that there is a well-established protocol that must be followed. And we know that there are laws to protect the people taking part in the study.”* 011-M.

“*So my work respects norms, is confidential and anonymous.”* 024-M.

With regard to ethics, 3 important concepts were mentioned: confidentiality, consent and transparency. The importance attached to confidentiality was particularly salient in the “mediators” group. The responsibility of everyone involved in the research to respect confidentiality was pointed out, as a guarantee of respect for the relationship of trust established with the interviewee.

“*Data is something that is created about someone or something. It has to be kept somewhere safe, no matter what form it takes, even if, for example, you've taken information about some house, even if it's not what you need. You can't leave it lying around...*” 024-M.

Informing the participant and obtaining his or her enlightened consent, an important element in good clinical practice, are key moments in the investigation of a participant.

“*You've got to have a way of speaking, you've got to be non-imposing in your speech, you've got to try and explain it to the person [...] You've come to see the person, so you normally want them to participate. Do you impose things on them? No... Somewhere along the process, you have to listen too. You have to listen.*” 024-M.

Transparency was cited in terms of the data collected (here in the context of hetero-administered questionnaires), the fact of showing the participant the questionnaire directly, or even suggesting that they mark the answers themselves, but also transparency in the use of the information and its possible final application. Respondents pointed out the crucial importance of transparency in the relationship of trust with participants, but also in not promising things that are impossible to deliver, or interventions that are not part of the research project.

“*Not only do you do the questionnaire and scroll all the questions in front of the person, so That they see exactly what they're answering, there's nothing else to do.*” 024-M.

“*But we do a survey, but in this survey, we can't tell people that we're going to help them find a residence permit, or get AME-CMU [health coverage]. That's abusive for me... Because it's not true.*” 004-M.

The concept of global ethics was also raised, as the aim of these procedures is to guarantee the rights of participants, and many people question their protocol aspect to the detriment of their actual effectiveness.

“*It's so interesting to understand the different conception of ethics, between the recommendation of the research world, and the ethics for the persons [from the target community]*” 025-R.

### Beyond the questions, a wide range of representation schemes to be made intelligible

Many of those interviewed mentioned the word “interface” to define the role of health mediators. Their action of linking, of bringing people together, takes place semantically, but also in culturally or socially determined differences.

“*Mediation is really a matter of interfacing between two different worlds, in this case a highly technical, highly qualified world of healthcare or healthcare research, and the beneficiaries or people, if we're talking about healthcare mediation in the strict sense of the term, or the people interviewed, selected or to be selected if we're talking about research.*” 020-R.

One of the barriers in intercultural research mentioned, between research teams and target populations during the interviews was language. In terms of language, while some researchers rely on their linguistic skills when working in the field, others are categorical about the impossibility of working without mediators. All the people interviewed, to varying degrees, underlined the importance of gaining an in-depth understanding of the respondents’ representations, in order to guarantee the quality of the answers to the questions asked and the quality of the data. What is more, given French Guiana’s linguistic diversity, literal translations are often difficult, and sometimes impossible. Many interviewees spoke of their own culture, a set of distinctive reading characteristics, as a prism of their understanding and inseparable from their person.

“*It's very important to understand the language in which you're doing the interview. I've said it clearly, I speak Portuguese because I was in Brazil, but when I find someone to do the interview in Portuguese, it's my colleague who does it, she's Brazilian, I could do it but it's my Brazilian colleague who's there, I want her to do it, it's much better. So the feeling goes better, it's much easier.*” 024-M.

“*Well, the point here is that in French Guiana, if you want to do a good survey, it's absolutely obligatory. You can't just go off on your own and say, "I'm going to ask people questions like that." First of all, there's the language barrier, and then there's the barrier of many other things.*” 003-R.

The notion of interculturality and schemes of representation was one of the most prevalent themes among the respondents. Differences in perception, where the majority of researchers are of Occidental origin, impact on the conduct of surveys, conceptual barriers, the weight on questions, redistributing the notion of intimacy and stigma with all the consequences that this implies.

“*The question was whether their last sexual encounter was transactional or not, and I find that a very intimate question. No problem answering it, even for me, a white, French doctor. On the other hand, to ask them if they use cocaine, well … I think there's an unbelievable reporting bias when it comes to that question. I wasn't expecting that.*” 005-R.

“*Beyond language, of course, there's a whole system of representational schemes and value systems that are built up and exist behind these languages. So, yes, I was talking about language, but it's the whole cultural understanding of these populations that can be sufficiently understood by certain researchers or certain research teams. Therefore the fact that it's more than just interpreting, it's really mediation that needs to be put in place more globally, in my opinion.*” 019-R.

Stigma extends to other aspects of populations: first of all, the populations surveyed may even be victims of discrimination linked to their conditions, such as migrants or illegal gold miners (populations that are the target of numerous research projects in French Guiana). Surveys have reported that study titles can also be stigmatizing for pathologies that are already heavy to carry, notably the definitions of a “case” and a “control,” and HIV.

“*People living with HIV, but there are two categories, but for me that's what gives people a little trouble. [...] It's people living with HIV who are taken as cases, but I thought that since the other diseases are part of sexually transmitted diseases, I thought they would reconsider the other pathologies, the other sexually transmitted infections as cases too. [...] but I thought this group of sexually transmitted diseases should be included in the group of cases.*” 007-M.

Differences in representations can also lead to differences in treatment, unequal relationships and power asymmetries, particularly with the medical profession. Many mediators also spoke of the importance of being legitimate and recognized in the departments in which they work.

“*The researcher in an intercultural situation is in the position of an outside observer. [...] For me, that's one of the traps you can fall into when you're working in interculturality, when you feel you're doing good, but the road to hell is paved with good intentions. We mustn't fall into any of the opposite excesses, and we mustn't be like the guys in their offices in Paris, who come and give us lessons and don't understand that things are different. At the same time, we mustn't take a paternalistic, "it's too complicated for them" view of things.*” 013-R.

However, for many of those interviewed, these cultural diversities are also cited as sources of openness and discovery, as a means of meeting people and creating links with them.

“*It's nice to see that, to see the diversity, to see the differences between the dishes. There are dishes that we don't know and that we can taste. It changes the flavors in your mouth, so it's important, it's interesting to see this culture.*” 022-M.

During the interviews, respondents reported that systems of thought, approaches and languages can sometimes differ even within groups, for example in the researchers’ group, people mentioned the case of cross-border collaborations, which can lead to difficulties in understanding. Community-based research was mentioned as one of the solutions to the different representations. For many of those interviewed, effective change in terms of health must come from the communities themselves.

## Second main theme: A profession sometimes misunderstood and underestimate

### The uncertain contours of this profession

Regarding the nomenclature around the intervention of mediators in research projects, various terms have been used, including interpreters, translators, investigators, surveyers, survey mediators, field agents, people from the community, community-based health workers and even sometimes informants, or knowers, without the nuance between the terms always being clearly spelled out.

“*They perfectly choose the word "facilitators" in English, “mediators” in French and community health-worker that's not health workers, but you know sometimes there are some nuances about this term.*” 025-R.

However, some of those interviewed described the difference between an interviewer and a mediator: the interviewer being in charge of neutral data collection, not proposing any intervention, and the mediator being more of a support person, aiming to help users with their care.

“*They weren't mediators, they were investigators - we trained them to carry out surveys, but they weren't mediators. They hadn't received any training in mediation, in terms of these surveys.*” 016-R.

However, the line between survey and mediation is a fine one, and depends above all on the design of the study in which people are involved. Sometimes, mediation skills are necessary within the framework of a survey, to facilitate the building of trust and acceptance. Interpreting and translation are components of the mediation profession, but some participants have gone so far as to contrast the concepts of interpreting and mediation.

“*In fact, because it's as if the mediator and the interviewer are getting a little closer together, but certain qualities aren't really highlighted [...] The mediator is very useful to the interviewer. Sometimes we try to juggle a little bit, I personally try to juggle a little bit to get an interview done.*” 023-M.

“*I'd put it more in opposition to interpreting, which is the strict translation of words, phrases or concepts. It's about adapting the discourse and semantics expressed by an individual.*” 001-R.

For the mediators in the field, the importance of giving sense to their work is a recurrent theme. Sometimes, the posture of investigator with no possibility of action is resented, as mediators are confronted with intolerable situations.

“*I'm almost moved and there are people who have nothing to eat, [...] the person isn't lying. It's finding I don't know,... working with other organizations, [...] even if we don't work as mediators. At least we can give... There are places where they give away food, free meals, but bring something. The person gives you an interview for 45 minutes, an hour sometimes 1h30 and without getting anything in return, to give a little something, even if it's not money.*” 023-M.

### Complex life journeys and an emotionally demanding profession

With regard to mediation work in research projects in French Guiana, the exposure of mediators to a high emotional charge has been reported on multiple occasions. Mediators are not always enough prepared or supported in their experiences. They are on the front line when it comes to the violence of life stories. In the context of their work, many reported the need to be able to relieve, even momentarily, the emotions of the people with whom they come into contact in their studies, as an extension of active listening. Many mediators also spoke of the importance of teamwork, so that they could talk to each other and unburden their experiences.

“*We also support people, and I mean mentally, because often the person tells you, tells you about their situation, and then you really have to do the work yourself to really make the person feel that they exist.*” 023-M.

“*06/22/2021: Meeting with [Name of mediator]: Good, but last week complicated: 3 people reported stories of rape: incest, rape from a young age. "The girls have forgotten about it". Very traumatic. "She felt like the guy was behind her". Voodooized. "No place to sleep, no family". "Let the woman express herself then don't cry with her".*” Notebook 1/1 - Guyasseremig Survey.

“*It's important because I think that when you talk, you take out of yourself what's inside, because if you keep it in, maybe when you get home [...]. You have this stress, this feeling of unhappiness deep inside you, and if you don't put it outside, you end up running into someone [...].*” - 022-M.

Mediators often come from the community and have themselves experienced a personal (migratory, human) journey, and their encounters with the people they investigate can echo their own experiences.

### Precarious mediation jobs

Whether in the field of healthcare or research, despite the importance of the presence of mediators, one of the factors highlighted is the lack of job guidelines for mediation positions. Project promoters have difficulty in financing positions, which are sometimes not even created. The contracts offered are often precarious, short-term and dependent on project funding. The limit to mediators’ involvement in research projects is primarily financial.

However, according to the people interviewed, as a result of a growing awareness of the key importance of these players, linked in particular to French Guiana’s health response to Covid-19, the situation is tending to evolve toward recognition of the profession. The number of active mediators, both in research projects and in the hospital context, is undersized in relation to needs.

“*It’s just unbelievable that in such a large hospital as Cayenne Hospital, mediation with such a wide range of populations, it’s unbelievable that we do not have a mediation service that’s more developed, even though I know there are people working in this direction.*” 010-R.

“*Now, these are not positions that are particularly valued. There is not really a mediator label in the French hospital service, so it’s a bit complicated, or at least there is not a type of contract that corresponds to their work, they are often on fairly precarious contracts, some act as mediators while having secretary, orderly, or hospital service agent contracts.*” 013-R.

“*Perhaps we could think about a status for these mediators who work on research projects, so that they have a real status and avoid precarious contracts. That they have a recognized place in research. And not end up being paid by the questionnaire...*” 016-R.

Another problem raised was the administrative situation of people recruited as mediators. Many of them come from communities and in the case of migrant populations and gold miners, for example, many are undocumented. This situation sometimes extends to mediators, whose short-term residence permits, or their non-renewal, are incompatible with training courses and work contracts.

“*Most of the mediators do not have papers, or they do have papers, but they are not yet stable, in fact, it’s a situation that... they themselves aren’t stable in their papers.*” 006-R.

### A balance between rigor and flexibility, relying on soft skills

When discussing health research projects, two binary issues emerged during the interviews, which can be seen as mirrors of each other: the duality between the scientific methodological aspect of a project and the reality on the ground, and the importance for mediators or anyone in the field, of demonstrating both rigor and flexibility to meet the study’s objectives. Inclusion sites are often specific, remote areas where people are far removed from care. The rigid, methodological side of the scientific framework is often set against the challenging, energy-intensive field reality faced by mediators. These two theoretical and practical aspects have been described as the two heads of the research hydra. The distance between these two aspects has been cited as one of the main difficulties in conducting research projects.

“*You know, working in the field isn't easy because there's rain, sun... There are bad people, there are dogs, but when you feel good in a place, when you're well treated, well welcomed, well received. It makes you want to do the job, to do it well and to keep on doing it.*” 022-M.

“*We'd been told "interviews" - "to do a survey", so it was really fixed on one term. But then when you get there, it's the survey, and you find yourself faced with a lot of unknown worlds, surprises, different things, and you really have to bring something to contribute. We have to make sure that we're able to do this work, to do the interview, but to do this interview we really have to try to press on each point, to press to pick and to really have the tools, to be able to do the interview.*” 023-M.

Mediators involved in research projects, and researchers alike, must constantly strike a balance between compliance with protocol and regulatory requirements, and the diversity of comprehension levels of their interlocutors, which requires the ability to adapt. Mediators are asked to fill in a questionnaire with precise questions for a variety of individuals, often with different priorities.

“*You have to be rigorous [...] but you also have to be flexible and resourceful, because nothing ever goes according to plan, so if you're too rigorous you'll never succeed. You have to find the balance between adaptability and flexibility, and still be rigorous so that the collection is homogeneous and serious. It's a balance that's not always easy to find.*” 013-R.

**Figure 2:**
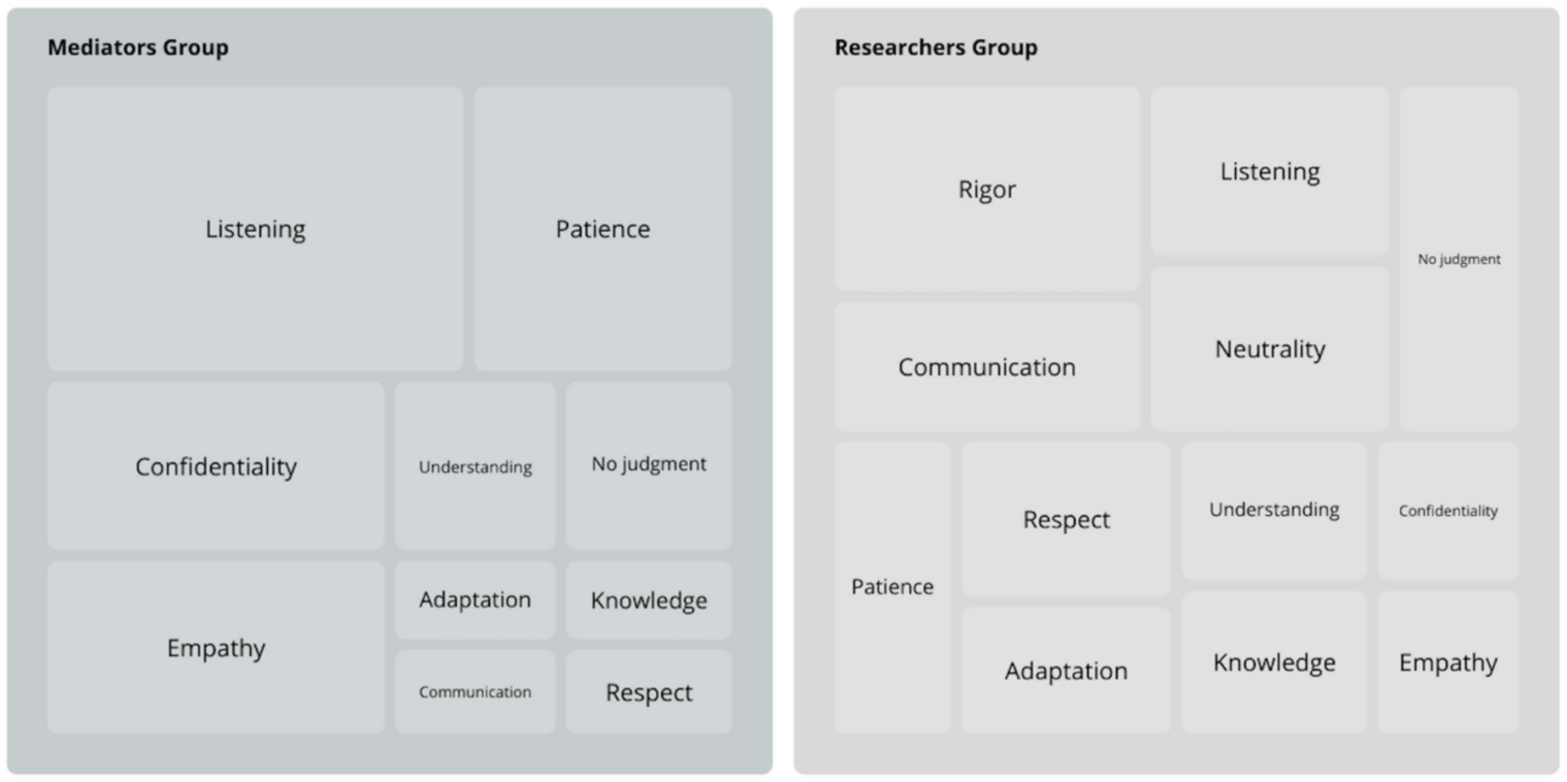
Distribution according to frequency of mention of the 12 qualities most cited at least five times and with propositional squares for the number of quotations per group as indispensable to the profession of health mediator by Inter-Med project respondents, by group.

The work of mediators in research projects relies heavily on soft skills. The duality between rigor and flexibility was apparent in these qualities, and the frequency with which they were cited varied between groups of respondents, as shown in Figure 2. For ease of reading, “knowledge of subject and protocol” has been summarized under the heading “Knowledge.”

Beyond soft skills, several other important elements emerged, in all groups. Firstly, the confidence and human qualities of the mediators are, according to the people interviewed, directly linked to the quality of the responses of the people included in a study, and therefore to the quality of the data. The time devoted to an interview was also raised as a quality factor.

“*This links up with interpersonal skills such as patience, the ability to really understand the role of neutrality, the ability to put people at ease, to avoid biased responses, etc. So it's a lot of interpersonal skills, motivation and the capacity to get involved in the project. After that, you need a minimum of organization, even if you can help them, after all, teamwork, that's for sure, and honesty, in fact, intellectual honesty.*” 026-R.

“*Because if you have no patience, you're always in a rush, and you do the interview and you're in more of a rush than the person... no, you can find answers, but they're not really answers*.” 004-M.

Along the lines of trust and data quality, the notion of honesty also came up, with several people sharing experiences where people in the field had falsified or even invented data.

“*I've talked about it with other people on other projects, and I've heard quite a few stories of people who, for various reasons, have gone so far as to falsify data*.” 026-R.

The notion of posture and knowledge of one’s limits has also been mentioned many times, and the soft skills that are so useful in the exercise of the mediator’s profession in the context of research can also serve less noble ends. The mediator’s position in a community can give him or her a kind of power that can lead to abuse. Finally, according to some researchers, it is also important to take a step back from the information transmitted by mediators in order to maintain accuracy in analysis.

“*It didn't go down well with the community and it's true that he had an attitude, he played the doctor a little bit, he had intimate relations with a member of the community who was in a relationship and so on. It was considered bad form. [...] really in a position to manipulate different people.*” 026-R.

“*And mediators don't necessarily know everything about their community. So there you have it... You also need to cross-check with book sources, and not buy everything the mediator says, which can also be wrong.*” 013-R.

## Third main theme: The specific context of research

### The world of academic research

Research, particularly hospital-based clinical research, is a standardized world with these codes. Many of those interviewed pointed to the importance of raising people’s awareness of research more generally, through widespread education and information. Many participants also expressed a desire to decompartmentalize knowledge.

“*For me “research” is not the scientist who have the knowledge, who only have the right to create knowledge. Knowledge is everywhere in every of us, and in the all learning that we have from the formal institutions and systems, and from the informal systems and all of them are very rich and all of them allow us to live together.*” 025-R.

The strong points of the research most frequently highlighted during the interviews were its methodology, the solidity of the results produced, their verifiability, as these results contribute directly to improving the health of the target populations. The scientific framework of the research also contributes to its value.

“*Research also means putting it into a framework, so that other people can do the same thing again and check that we're finding the same information. [...] All this framework and all these common codes of science, which for me... These are the strengths of research.*” 026-R.

The most frequently cited limitations of the research are the distance between theory and reality in the field, the extensive and under-valued logistics of the projects, biases (at all stages, cultural, declarative, at the time of sampling or subjectivity), the lack of time, the regulatory aspect and the lack of funding (or its oriented nature on certain subjects). Another element that emerged as one of the difficulties of the research was the complex and imperfectly human nature of people’s relationships with each other (through collaboration, personal egos, international relations or management).

“*Like all human constructs, [Research] is subject to our imperfect organization, the need to find credits, to find funding to drive it forward. Having to deal with ego wars, the frustrations of researchers, research administration etc…*” 013-R.

“*It's really the limits of research, the limits of research is that you always find the same types of profiles in research who have done the same studies, who have the same way of thinking.*” 026-R.

The perception of the limits and strengths of research, and the desire for improvement, coincide with the scope of each person’s professional reality. Thus, in the researcher’s group, where professions are highly specialized, everyone’s vision is “profession-centric.” In the “mediators” group, all visions converge to a much greater degree, with the well-being of users being a predominant common objective.

“*When you're in charge of a team, it's hard to understand, because people have families, they have to carry loans, and if we don't get the boat to the right place, they'll lose their jobs. So, we're dictated by imperatives that are probably not the right ones, and the processes we're working on don't take enough account of the diversity of what humanity is, particularly in terms of its perceptions.*” 001-R.

In this context, interdisciplinarity is of prime importance, leading to mutual openness. The collaboration between researchers and mediators has been very positive, and has led to an evolution in vision: a bigger world.

“*The vision, yes … it's evolved because … the way I thought I was going to work, in fact, it's more vast […] There are quite a few surprises, it's changed me. There's a breadth to it, if I can say that. In fact, the discoveries are enormous.*” 024-M.

“*Working as mediators, in fact, on research which, as I was saying, is still very vertical, and now we're talking more and more about participative research, and the decolonization of knowledge, etc., which opens up the mind.*” 026-R.

### Peer concepts and differences in perception

Three definitions were explored in the interview guides during the survey: data, sample and representativeness. For the definition of datas, all respondents in all groups met the definition of academic research, i.e.: the results of observations or experiments carried out on a subject, used as part of a research project, which can take several forms. The majority of those interviewed said they attached great importance to data, as the basis of their research projects. Others, fewer in number, took a more nuanced approach to importance, warning of the biases associated with datasets and their analysis.

“*[Data] so I give it a lot of importance, provided I know what we're talking about and where it comes from.*” 001-R.

All participants in the “researchers” group met the definition of a sample given by academic research, i.e., a set representative of a population with the same characteristics, part of the population of interest. However, half the participants in the “mediators” group reported an academic definition, and half mentioned a broader definition not limited to the research world, or some other perception.

“*It's a sample, it's a part, it's a little parcel, that's all. Of an original piece, to really give, to give an idea to others. I've taken this sample to go and buy the same product, for example, or to try and find the same product, or a more or less similar product, that's close to the original product.*” 023-M.

Finally, questioning the concept of representativeness showed two very different perceptions depending on the group. The extreme majority of participants in the “researchers” group gave an academic definition, specific to research, of representativeness, i.e., the ability of a sample to correspond to the target population according to defined criteria. None of the participants in the “mediators” group gave this definition. Instead, they spoke of “representation”, of their posture, and of carrying the image of the institution for which they worked. Wearing this image is demanding, and sometimes brings hard feelings. Indeed, a number of mediators reported having been victims of violence during the investigations they conducted, because they were assimilated to the institution’s representatives (notably the hospital for which they worked) and were at that point the only available interlocutors.

“*Traumatic with the hospital and unfortunately, it's just us … We're the ones who have to absorb everything […] It was a man who attacked me like that. It was a gentleman who told me … I introduced myself, and the gentleman said, "Oh yes, yes, it's the hospital in Cayenne, that's put me in this state, get out! And I was on the 4th floor, and the gentleman was coming out of his apartment, and I couldn't even move.*” 023-M.

### Health research: a matter of purpose

Research was defined by respondents as a process of creating knowledge, increasing expertise in a particular field, or on a particular population. The question posed must be relevant, with a valid methodology for answering it. The purpose of health research is to understand past, present and future phenomena. However, the definition of research has never been dissociated from its aim: to improve a situation, or the health of a population.

“*Generally for me, these are things we want to know, then research to see what we can contribute, what we can improve, or what we can... When there's a problem, we do research to see where the problem exists and what solutions we can come up with.*” 007-M.

Health research was even contrasted by some respondents with fundamental research, whose practical purpose was not pre-established. A number of respondents pointed out the importance of questioning this purpose before setting up projects, as they felt that some projects served medical careers more than the target populations.

“*And this, I think, is really an essential ethical element to take into account: why do we do research, or is it just for publication or personal interest, or is there a real benefit and operational consequences for the beneficiaries?*” 020-R.

For many respondents, the definition and understanding of this research aim needs to be clearly explained to participants, in order to justify the questions asked, and sometimes their intimate nature. However, this explanation must be completely transparent. According to many respondents, outside the framework of interventional research, it is important not to promise immediate changes to the study population, but to explain the process of data creation, which can then be used for action.

“*The sense is to create knowledge which would be beneficial for the health community, what I feel is that we need to be very honest when we ask people participating in research, on how we can relieve… be beneficial in that*” 025-R.

**Figure 3:**
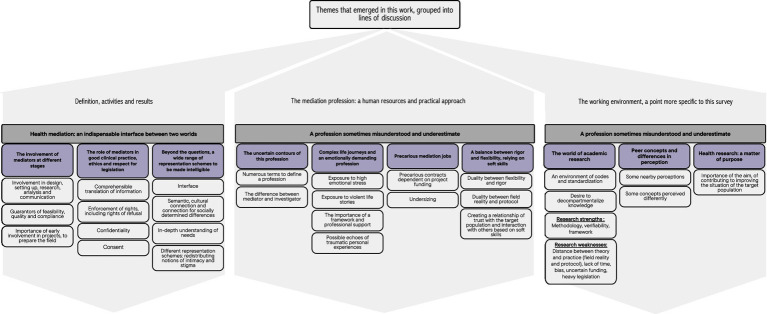
Main results: perceptions, attitudes and opinions on research of mediators and researchers collaborating on research projects; strengths and difficulties encountered during this cooperation, in French Guiana in 2022 and 2023.

Finally, always with an eye to “purpose” and finality, questioning the interest of the studies for the target populations is important in the design of a study, and it is important to think about the restitution of the information collected to the population of interest (Figure 3).

## Discussion

In terms of respondent profiles, for the researcher’s group, all respondents were born in the West and come from high-income countries. In this group, too, people have had long studies, and more than two-thirds have an academic level higher than a doctorate. As for the mediators’ group, the majority of those who took part in the survey were born in South America, one in French Guiana (France), one other in Brazil (an upper-middle-income country), but the majority of respondents in this group come from Haiti, one of the world’s poorest countries. In the mediator’s group, while all of the respondents had completed a high school diploma, only half had gone on to university. The biases of conception and interpretation inherent in the academic and Western backgrounds of the researchers are also likely to influence the research and interpretation of the results. In the field of research, power asymmetries are deeply rooted and have already been described, particularly in global health partnerships, between researchers from high-income countries and those from middle-income and especially low-income countries (often the places where research is carried out) (17, 18). What is more, the epidemiological distribution of health issues in French Guiana means that many research projects target people with low levels of health literacy, most of whom are racialized, of foreign origin and living in precarious conditions. These elements raise a color line superimposed on the relations of power (and therefore hierarchy) in our post-colonial societies.

The participation of mediators at different levels of the survey was described as important. First of all, the question of bias comes up, as studies carried out with foreign-gaze only depict part of the reality (17). In clinical research, their intervention helps to reduce ethnocentrism and negative stereotypes, and thus to provide quality data. Data quality and reliability is one of the pillars of good research practice (19). Information and consent are key points in good clinical practice, and in the Jardé law, which defines the framework for research in French territory (16, 20). According to the World Health Organization (WHO), quality research involves, in particular, the quality of the questions asked, the quality of the design and conduct of the project, and the communication of results (21). In the development of these elements, mediators who know the terrain and the community have a role to play. Beyond their involvement in the design, the importance of their intervention beforehand, to familiarize the target population with the project, and the researchers with the population, was mentioned. These elements are also in line with the WHO Code of Research Ethics, which refers to respect for people and communities (21). The involvement of peers and people from the community, and even the development of community-based research to question the feasibility, acceptability and suitability of research projects, has repeatedly demonstrated its effectiveness in healthcare (14, 22, 23).

Ethics are an intrinsic part of any research project involving human subjects. Obtaining ethics committee approvals and the regulatory aspect of a project, were cited as a burden by people in the researcher’s group. The perception of ethics approval procedures as an “obstacle” to be overcome is not isolated (24). The Declaration of Helsinki and the Nuremberg Code aim to protect participants in research involving the human person and to structure the ethical approach around respect for people, health and justice (25–27). Beyond regulatory approval and good clinical practices guaranteeing the quality of a study, ethics is a question that must be asked at every stage of research, particularly in environments where power asymmetries may exist (24). Some French Guianese people, far from the coastal areas, maintain a traditional way of life, often far removed from the written, quantitative or computerized systems of the West. However, these people may also be concerned about the confidentiality of their health information (especially in the case of a stigmatized pathology). These differences of vision can be a source of incomprehension and even concern for the populations targeted, and above all can imply inefficient application of the rights of study participants. Mediators have a role to play in transforming this regulatory framework into a protection of individuals. Through interpretation, explanation and time, they guarantee informed consent, respect for and understanding of users’ rights. The Declaration of Helsinki reminds us that anyone potentially involved in research must be properly informed of the objectives, methods, funding, expected benefits, risks and any other relevant aspects of the research. The notions of confidentiality and informed consent are also part of this declaration (25). Lastly, transparency (from data collection to the final outcome), in addition to contributing to the creation of a link of trust between interviewer and interviewee, is one of the points of commitment to clinical trials put in priority by the WHO (28).

Mediators act as an interface between the academic, scientific and protocol-based world of research, and the reality of life of target populations. This role can be played by translating concepts such as legal ones, but also by translating cultural concepts. Beyond language barriers, conceptual barriers and differences in representation schemes play a particularly important role in bringing together two very different populations. This aspect is essential in French Guiana. These elements also remind that culture is inseparable from the individual. Ignoring these differences can lead to disrespect through ignorance. In a survey, these differences redistribute the notion of intimacy, and the perceived weight of questions may diverge between the designers of a questionnaire and the people to whom it is addressed. A large number of social science studies have been calling for these differences in approach to be taken into account, and for the limitations of a purely biomedical approach or the inadequacies of institutions inspired by the scientific and technical model of medicine to be overcome (29). More globally, the WHO is calling for a “One Health” approach that is multisectoral, multidisciplinary, societal and involves the environment (30). These differences in perception highlight differences in perception of taboo and stigma. Stigma can affect target populations and is directly linked to their health problems, particularly in the case of migrants and illegal gold miners. The way in which the population is viewed by society, and the political choices made as a result, can have an impact on their health. Racism and stigma, and their consequences on individual socio-economic situations, are both proven sources of inequality in terms of health (31). Lastly, the titles of the studies themselves can be stigmatizing, written through a medical or epidemiological prism, reinforcing the burden of representations carried by certain individuals, particularly people living with HIV, who are still heavily exposed to this weight, including in the care environment (32). In this configuration, socio-cultural differences can lead to power asymmetries, differential treatment. Some works call for the need to question the essentialism, without rejecting efforts to understand the relationship between origin, culture and health, and to relativize these concepts in order to arrive at alternative useful and applicable formulations in the field of health and health care (33). And last but not least, the mixing of different individuals, and confrontation with diversity, can be a source of open-mindedness and learning.

The title of the position itself is subject to different names, and conceptions in French. In English, the word “facilitator” is the most commonly used, although this is not a literal translation of the word “mediator.” This highlights a conceptual barrier between French and English, in terms of vocabulary. In these different studies, by their mission, the role of the mediators can be described as halfway between investigators and community health workers (34). There is a fine line between mediation (social and health work, human and cultural skills) and investigation (neutral, scientific, systematic and non-interventional). Investigators are immersed in environments where social and health needs are very present. The weight and mental consequences of exposure to contexts of illness, pain have been widely highlighted (35–37). Sometimes, the impossibility of action is particularly difficult to live with. Experiencing this combination of listening and powerlessness in health care, and its link with the status of the listener, requires self-knowledge, emotional resources and a confrontation with renunciation (38).

Listening to and being exposed to stories of trauma can have serious consequences for the health of those who receive them. Surveys in French Guiana are concentrated in vulnerable populations, exposed to hunger, physical and sexual violence, and to situations of great insecurity (39–42). Mediators are on the front line. Many cases of vicarious trauma have been described, among caregivers exposed to situations of violence (35–37, 43). Some of the mediators themselves have experienced difficult life situations, many do not have permanent residence permits, and some may be exposed to life situations similar to those they are investigating. Team support is absolutely essential, as accompaniment, training, psychological supervision and social and professional support have already demonstrated their positive impact on caregivers’ mental health (44, 45).

Despite the importance of mediators in projects, their positions are often precarious, short-term or not well-defined. The fact that the profession is undersized in relation to needs adds tension to an already demanding job. The question of opening positions and obtaining funding is one of the limits to their involvement early in projects. In practice, the importance of ensuring the long-term viability of these positions, in particular through the definition of their role and the duration of their funding, has already been highlighted (46). More generally, this question of sustainability is addressed by the health institution Santé Publique France, for whom one of the steps to be taken to facilitate the development of mediation on a large scale and guarantee the conditions for its effectiveness, is the question of securing jobs (47).

The duality between rigor and flexibility was highlighted, as a mirror of the duality between the scientific and methodological aspect of a project, and the reality of the field in which it is implemented. For the studies mentioned, these two inseparable elements are based on collaboration between mediators and researchers, and are illustrated by the importance attached to soft skills. Listening, for all groups, was mentioned as central. The link between active listening and adherence to a protocol is intuitive and described (48). The correlation between good communication, continuity of care, patient safety and the efficient use of resources, time and funding has been amply demonstrated (49). Different perceptions exist: on the researchers’ side, rigor, neutrality and non-judgment were highlighted, suggesting the academic aspect and the particular importance attached to data collection. As for the mediators, they highlighted patience, empathy and confidentiality, suggesting the particular importance attached to the well-being of the participants. These elements can be interpreted as the two cohabiting faces of health research.

This study deals specifically with research taking place in a hospital context that is particularly standardized. Interdisciplinarity brings many positive evolutions to project management. In this environment, or in any relationship, the complexity of human nature is expressed, multiplied by differences in beliefs, attitudes and norms that are often unconscious (50). In intercultural collaboration, strategies such as identifying expectations, adapting, monitoring attitudes and moods, considering stereotypes, developing knowledge bases and skill areas, suspending judgment, can be beneficial and facilitating (50). The strengths of research are defined as its methodology, making it possible to create solid results that contribute directly to improving the health of the target population. Reliability and validity are the historical strengths, the foundations of research in all fields (51–53). The weaknesses mentioned—the distance between theory and practice, underestimation of logistics, bias, lack of time or money—are identified and usual in many research projects (15, 54–57).

As mentioned above, differences in perceptions and norms can be expressed in cross-cultural collaboration. Here, the definitions of sample, data and representativeness suggest that part of the vision of the research world is shared between researchers and mediators, and that some of their realities are not superposable. Observing and decoding differences in a given situation, and building common frameworks (e.g., shared glossaries, where one definition does not take over another), can be valuables contributions that make collaboration more fluid (50). Lastly, mediators in the field may be exposed to resentment against the institutions they represent, and this factor should encourage to consider the communication around projects in order to guarantee their safety, and more generally to question the mistrust that exists in relation to these structures.

According to the Oxford Language Dictionary, research is defined as the detailed study of a subject, particularly with a view to discovering new information or reaching a new understanding. In this study, the proposed definitions are in line with this, but place the focus one step further on action, and the particular need associated with health research: to improve the health of the populations concerned. According to the Société Française de Santé Publique, the distinctive feature of public health research is that it indirectly includes a perspective of action, or decision support (58). However, despite this optic of action, improving the health of populations depends directly on the political choices that will be made following communication of the results. As current events in France have shown, with the proposed abolition of state medical aid (health coverage for primary care for undocumented migrants in France), or the regular questioning of the right to abortion throughout the world, despite the accumulation of scientific evidence and international recommendations, political decisions can be driven by other motivations, to the detriment of the health of populations (59–61). The World Health Organization’s Code of Conduct for Responsible Research states that research must be based on people’s health needs, as well as on all factors likely to influence quality of life (21). In practice, in France, the ratings associated with publications play an important role in budget allocation for healthcare establishments, and in the development of individual careers, influencing the orientation of investigations and questioning the ultimate usefulness of their results.

### Strengths and limitations

This study has a number of limitations: first of all, it is an analysis limited to the French Guiana experience, the territory of French Guiana being specific in many aspects. The last part of the results is linked to the Guianese context, hospital-based, and less transferable than the first two. Another limitation is that the definition of the profession of mediator here is non-specific, open-ended and covers different definitions. The interviews carried out involved a variety of themes, and as the projects touched on a variety of subjects, only a general and not very in-depth analysis was possible for certain themes. Finally, the volume of information was substantial, and the analysis involved setting limits, coding concepts and choosing definitions, with the biases that this implies. What is more, this work attempts to describe a complex, constantly evolving system for which few works or conceptual frameworks exist. One of the limitations of this study is that a large proportion of the studies discussed during the interviews were cross-sectional epidemiological studies, based on hetero-administered questionnaires. Qualitative studies and intervention research were also mentioned, but to a smaller degree.

Despite peer review, the analysis was mainly carried out by a Western-born, medically-trained, from the world of academic research, who had been involved in coordinating health research projects: a social, cultural and linguistic bias cannot be excluded. Another bias to be taken into account is that, in the sample collected, the group of researchers was larger than the group of mediators, which could influence the discourse collected, giving more weight to the views reported by the group of researchers. What is more, as mediators are an integral part of research teams, here the definition was made in two blocks, taking the risk of placing two arms of the same body in opposition. Finally, throughout this study, the notion of community-based research came up, although the projects carried out did not fall into this definition.

Despite its limitations, this study also has a number of strengths. The first of these is its internal questioning of the way research works, highlighting a willingness to understand and look critically at the endogenous and exogenous creation of knowledge. To our knowledge, this is the first analysis carried out in French Guiana of the perceptions, attitudes and opinions of mediators and researchers on the participation of mediators in clinical research, and on the context of interculturality. This analysis covered several perceptions, involving mediators, researchers, people working on several projects, and mutualizing a wide range of experiences. The interviews were supplemented by the exploration of 2 field experiences extending over 2 years, grounding this analysis in practice and reinforcing its quality and robustness. Despite the fact that some aspects were only explored on the surface, there is a strong convergence of perceptions on many themes, both within and between groups. Despite the diversity of the projects, the study highlighted some particularly salient and redundant themes. As the French Guiana experience is highly diversified, despite the specific nature of this territory, it is possible to presume, given the plurality of the populations involved, that the themes, learnings, conclusions and recommendations may be transferable to similar situations, particularly in the context of epidemiological health research in an intercultural context. Although the studies mentioned above do not fall into the definition of community-based research, they can be likened to participatory research, and were driven by the same values, the same philosophy, and the same desire to decompartmentalize knowledge and empower populations. And, last but not least, this study offers an important perspective on healthcare and research actors in development, and opens up discussion on key themes where relatively little literature exists. This analysis, although based on experiential knowledge, was supported by existing conceptual frameworks, particularly with regard to intercultural skills. This analysis was reviewed by people with a variety of skills (mediators, anthropologists, researchers) to limit bias and reinforce its internal validity.

## Conclusion

This study touches on a number of theoretical and practical aspects of the development and implementation of research projects. Crossed all the way through by respect for good clinical practice, respect for rights and individuals, transparency and data quality, it questions the power issues in place along projects and challenges ethnocentrism. Particular attention must be given to the weight of words and the biomedical prism in studies, to avoid adding the weight of a new stigma to an already heavy burden. Mediators play an important role in creating a link of trust, which is essential to any collaboration, in building acceptance among the populations concerned, and in guaranteeing the quality of the data collected. Their involvement is a guarantee of quality, and must take place all along the project, but also early on, with a view to familiarization. Mediators act as interfaces between two very different worlds, translating languages and concepts. The work of mediators is based on soft skills, and is carried out across the abyss between rigor and adaptation, theory and practice. The creation of common tools, and improved attention to communication among teams, would enable us to better navigate between the demands of protocol and the reality of the field. Language, thinking, relationships with the body, health and perceptions of others are all part of a complex system: the individual, and research must do everything in its power to respect the individual to the greatest possible extent. The presence of mediators is an opportunity to transform heavy administrative procedures into the application of ethics and the protection of people along the whole project. Because of the populations they work with, mediators are highly exposed to mental health issues in their emotionally demanding profession, and need to be supervised, protected and supported. Despite their importance, these professionals are often employed in precarious positions, or experience professional instability. Structuring and securing the profession would contribute to its recognition. This study also reminds us of the importance of promoting the decolonization of knowledge, and participatory research, in the interests of transparency and adequate response to healthcare needs. Before any research project is launched, its ethical and impartial aspects must be taken into account, and its purpose and possible impact on the health of the target population must be transparently questioned. Finally, from a participatory research perspective, this study shows that the full integration and recognition of mediators in research projects could be beneficial in research carried out in an intercultural context.

## Data availability statement

The original contributions presented in the study are included in the article/supplementary materials, further inquiries can be directed to the corresponding author.

## Ethics statement

The studies involving humans were approved by the following ethics committee: Comité de Protection des Personnes (CPP) Sud-Est I under CPP number 2021–119. The studies were conducted in accordance with the local legislation and institutional requirements. The participants provided their written informed consent to participate in this study.

## Author contributions

LA: Conceptualization, Data curation, Formal analysis, Investigation, Methodology, Project administration, Resources, Supervision, Validation, Writing – original draft. M-AT: Formal analysis, Investigation, Methodology, Supervision, Validation, Writing – review & editing. MO: Methodology, Supervision, Validation, Writing – review & editing. AA: Funding acquisition, Methodology, Project administration, Resources, Supervision, Validation, Writing – review & editing. NV: Funding acquisition, Methodology, Resources, Supervision, Writing – review & editing.
